# Case Report: a novel *PNPLA2* homozygous frameshift variant causing severe neutral lipid storage disease with myopathy (NLSDM) in a Moroccan patient

**DOI:** 10.3389/fgene.2026.1701218

**Published:** 2026-05-26

**Authors:** Elena Faedo, Mary Marcela Araujo Chumacero, Sara Missaglia, Ariane Lunati-Rozie, Gianmarco Severa, Marion Onnée, Bornale Das, Eleonora Martegani, Noemie Lafage, Lina El Bejjani, Ines Barka, Stéphanie Gobin-Limballe, Bouchra Badaoui, Jean-Pascal Lefaucheur, Daniela Tavian, Edoardo Malfatti

**Affiliations:** 1 Reference Center for Neuromuscular Disorders, APHP Henri Mondor University Hospital, Créteil, France; 2 Department of Neurosciences, Rehabilitation, Ophthalmology, Genetic and Maternal and Infantile Sciences (DINOGMI), University of Genoa, Genoa, Italy; 3 Hospital Nacional Edgardo Rebagliati Martins, Lima, Peru; 4 Laboratory of Cellular Biochemistry and Molecular Biology, CRIBENS, Catholic University of Sacred Heart, Milan, Italy; 5 Department of Psychology, Catholic University of Sacred Heart, Milan, Italy; 6 University Paris Est Créteil, Inserm, U955, IMRB, Créteil, France; 7 Université Paris-Est Créteil Val de Marne, Créteil, France; 8 Hôpital Universitaire Necker-Enfants Malades, Paris, France; 9 Clinical Neurophysiology Unit, Henri Mondor University Hospital, AP-HP, Créteil, France; 10 EA 4391, ENT Team, Paris-Est Créteil University, Créteil, France

**Keywords:** Jordans’ anomaly, lipid droplet accumulation, neutral lipid storage diseases, neutral lipid storage disease with myopathy, *PNPLA2* gene

## Abstract

Neutral lipid storage disease with myopathy (NLSDM) is an ultra-rare autosomal recessive lipid metabolism disorder caused by *PNPLA2* variants, leading to defective adipose triglyceride lipase (ATGL) function and pathological triglyceride accumulation in multiple tissues. Fewer than 150 cases have been reported worldwide, and the clinical spectrum remains incompletely defined. A 57-year-old Moroccan man presented with diabetes, hearing loss, cataracts, hypertrophic cardiomyopathy, and asymmetric weakness. Whole-body muscle MRI revealed severe fatty infiltration of the supraspinatus muscles in the upper limbs and asymmetric right biceps femoris and left lateral gastrocnemius muscle with a patchy pattern in the lower limbs. A deltoid muscle biopsy revealed vacuoles filled with lipids. Peripheral blood smear analysis showed Jordans’ anomaly. Next-generation sequencing disclosed the novel homozygous c.1043del, p.Phe348SerfsTer18 *PNPLA2* frameshift variant predicted to disrupt the adipose triglyceride lipase (ATGL) patatin domain. Bioinformatics protein modeling predicted disruption of the patatin domain and global structural instability, likely abolishing enzymatic activity. Western blotting performed on patient-derived muscle tissue revealed a truncated ATGL protein, functionally validating the truncating effect of this novel variant. This case broadens the phenotypic and molecular understanding of NLSDM.

## 1 Introduction

Neutral lipid storage diseases (NLSDs) are rare inherited metabolic disorders characterized by accumulation of lipid droplets rich in triglycerides (TGs) in the cytoplasm of various cell types and tissues (Fischer et al., 2007). The underlying defects impair the first step of intracellular lipolysis, where triacylglycerol (TAG) is hydrolyzed into diacylglycerol (DAG) and fatty acids (FAs). This process is catalyzed by adipose triglyceride lipase (ATGL), which requires activation through its interactions with α/β hydrolase domain-containing 5 (ABHD5) (Lass et al., 2011). Dysfunction of these enzymes leads to insufficient FA mobilization, leading to the pathological accumulation of intracellular TAG (Fischer et al., 2007; Gluchowski et al., 2017).

Variants in *ABHD5* gene [also referred to as comparative gene identification-58 (*CGI-58*)] are responsible for NLSD with ichthyosis (NLSDI or Chanarin–Dorfman Disease, OMIM: 275630) (Dorfman et al., 1974; Chanarin et al., 1975; Lefèvre et al., 2001). In contrast, *ATGL* gene [also called patatin-like phospholipase domain-containing 2 (*PNPLA2*)] variants lead to NLSD with myopathy (NLSDM) (Fischer et al., 2007).

NLSDI typically presents with non-bullous congenital ichthyosiform erythroderma, hepatomegaly, and multisystemic involvement such as hearing loss, ataxia, cataracts, intellectual disability, microcephaly, and intestinal involvement with mild myopathy (Pennisi et al., 2017).

NLSDM is characterized by childhood- or adulthood-onset myopathy and cardiomyopathy, often with hepatomegaly, diabetes, chronic pancreatitis, and short stature (Zheng and Liao, 2018), without ichthyosis and central nervous system (CNS) involvement. Muscle biopsies show atrophy, fibrosis, and lipid accumulation in type 1 fibers, with frequent rimmed and autophagic vacuoles (Chen et al., 2010; Kaneko et al., 2014). Asymmetric muscle weakness, often predominant on the right side, primarily affects the proximal muscles of the arms, sometimes accompanied by scapular winging, and the proximal and distal leg muscles (Samukawa et al., 2020). Cardiomyopathy—including both dilated and hypertrophic forms—has been described in approximately 40% of patients and may progress to heart failure, occasionally necessitating cardiac transplantation (Rajani et al., 2020). Notably, extensive TG accumulation has been identified within cardiomyocytes and coronary artery walls (Hara et al., 2023).

NLSDs are extremely rare conditions, for which natural history remains poorly understood, and to date 132 NLSDM patients harboring 72 different *PNPLA2* variants have been reported (Lefèvre et al., 2001; Fischer et al., 2007; Missaglia et al., 2015; Yang et al., 2024).

Here, we thoroughly describe a patient with NLSDM associated with cardiomyopathy, diabetes, and hearing loss, linked to a novel *PNPLA2* pathogenic variant resulting in a truncated protein.

## 2 Case description

A 57-year-old man, born to healthy consanguineous parents from Morocco, manifested with insulin-dependent diabetes at 33 years, which was attributed to pancreatic dysfunction, followed by right-sided sensorineural deafness. The patient developed moderate and bilateral non-proliferative diabetic retinopathy (NPDR) at 48 years, followed by a cataract. Cardiologic workup at 48 years revealed hypertrophic cardiomyopathy. Cardiac magnetic resonance imaging (MRI) showed asymmetric left ventricular hypertrophy (maximum septal thickness: 21 mm), mild right ventricular hypertrophy, and a mildly reduced left ventricular ejection fraction (LVEF: 49%). Electrocardiography (EKG) revealed anterolateral ST-segment abnormalities. Subsequent coronary angiography identified diffuse atheromatosis, including a 50% mid-left anterior descending (LAD) artery stenosis and critical stenosis of a diagonal branch, both managed medically. A first obtuse marginal branch stenosis was treated with drug-eluting stent implantation. His neuromuscular symptoms started at 47 years with pain in the right shoulder, followed by progressive difficulty elevating the right arm and walking difficulties with frequent falls (Table 1).

**TABLE 1:** Timeline of key clinical events in the patient’s disease course. The table summarizes the age at onset and nature of major manifestations, including metabolic, neurosensory, cardiac, and muscular symptoms, highlighting the progressive and multisystem involvement characteristic of NLSDM.

Patient’s age	Clinical event
33 years	Onset of insulin-dependent diabetes
40 years	Right-sided sensorineural hearing loss
47 years	Right shoulder pain and progressive asymmetric weakness
48 years	Non-proliferative diabetic retinopathy and cataracts
48 years	Diagnosis of hypertrophic cardiomyopathy and coronaropathy
55 years	Gait impairment and frequent falls

**FIGURE 1:**
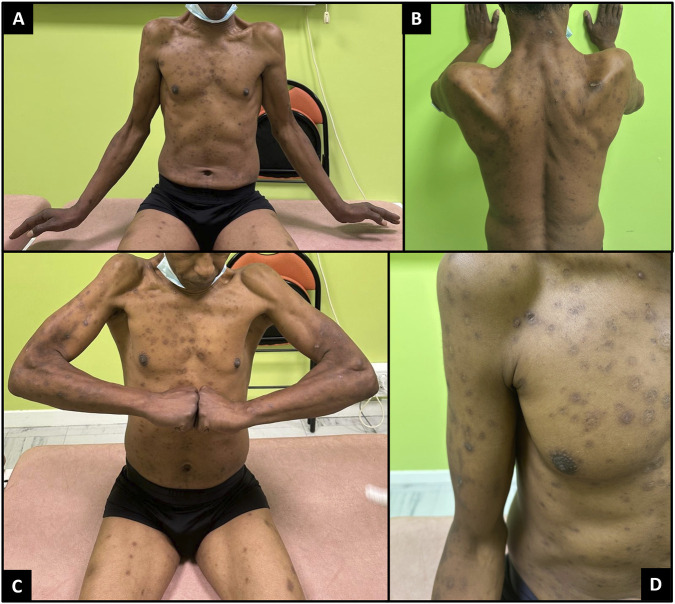
Patient features. **(A,C)** Limited abduction of the upper limbs at 30°. **(B)** Scapular winging with muscle atrophy of the scapular girdle predominant on the supraspinatus and infraspinatus muscles. **(A–C)** Atrophy of the scapular girdle. **(D)** Target-like urticarial lesions on the chest and torso, consistent with prurigo.

Neurological examination at 56 years showed a waddling gait (Supplementary Video S1) and marked asymmetric muscle atrophy of the scapular girdle, notably the *supraspinatus* and *infraspinatus* muscles (Figure 1B), and bilateral atrophy of *gastrocnemii* was also observed. Manual muscle testing showed symmetric arm elevation and abduction limitation to 30°–45° (Figures 1A–C) and deltoid (3/5 MRC), right bicep (4/5 MRC), hip flexor (4/5 MRC), and gluteal (4/5 MRC) muscle weakness. Deep tendon reflexes were absent. Physical examination disclosed the presence of target-like urticarial lesions predominantly affecting the chest and torso, which is consistent with a prurigo (Figure 1D); a previous cutaneous biopsy performed in another hospital was non-contributory, showing only unspecific inflammatory changes. The serum creatine kinase levels were 1,859 U/L (normal value < 230 U/L).

## 3 Diagnostic assessment

**FIGURE 2:**
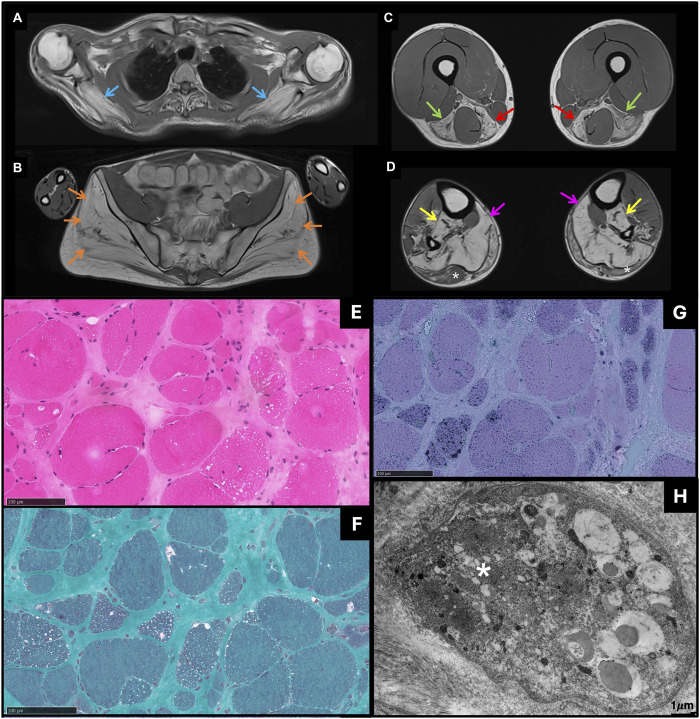
Muscle MRI T1-weighted sequences of limbs and myopathological findings in skeletal muscle. **(A)** Symmetrical fat substitution of supraspinatus muscles (blue arrows). **(B)** Symmetrical fat substitution of the gluteal muscles (orange arrows). **(C)** Asymmetrical involvement of the semimembranosus (green arrows) and long head of the biceps femoris (red arrows) and sparing of interposed semitendinosus muscles. **(D)** Complete fatty substitution of the tibialis posterior (yellow arrows) and soleus (pink arrows) muscles. Asymmetric involvement of gastrocnemius lateralis muscles with patchy features (white stars). **(E–G)** Light microscopy images (scale bar = 100 µm) acquired with a NanoZoomer® S360 digital slide scanner. **(E)** Hematoxylin and eosin (H&E) staining shows marked myofiber size variation, splitting of fibers, and the presence of variably sized vacuoles. **(F)** Gömöri trichrome staining showing the presence of vacuolated fibers and highly increased connective tissue. **(G)** Sudan Black staining showing the presence of augmented lipidic content and vacuoles filled with positive material. **(H)** Transmission electron microscopy (TEM) images (scale bars = 1 µm) acquired with Tecnai T12 showing an atrophic fiber with a disarrayed sarcomeric structure and numerous large electron-lucent lipid droplets surrounded by an empty area (yellow arrow) and containing redundant vesicular autophagic material with variably sized, electron-lucent lipid droplets (star) and organelles.

Whole-body muscle MRI demonstrated diffuse fatty infiltration, graded as Mercuri (Beltrame et al., 2014) stage IV, in the *supraspinatus* muscles bilaterally (Figure 2A), as well as in several lower-limb muscles (Figures 2B–D). There was marked symmetrical involvement of the middle and small gluteal muscles (Figure 2B), the upper portion of the *gluteus maximus* and *semimembranosus* muscles (Figure 2C), posterior tibial and soleus muscles, and the medial head of the *gastrocnemius* (Figure 2D). Fatty replacement was asymmetrical in the biceps femoris—stage IV on the right and stage III limited to the distal portion on the left—and in the lateral head of the *gastrocnemius*—stage IV with a patchy distribution on the left and stage II on the right (Figure 2D). The anterior compartment muscles of both the lower legs were symmetrically involved to a lesser degree (stage II).

Peripheral blood smear revealed Jordans’ anomaly, with characteristic punched-out vacuoles in all leukocyte types, including rare granular precursors and basophilic lymphocytes (not shown).

A left deltoid muscle biopsy performed at 56 years showed myopathic features, including fiber size variation, prominently atrophic muscle fibers, myofiber splitting, and internalized nuclei (Figure 2E), along with highly increased endomysial and perimysial connective tissue (Figures 2E,F). Both atrophic and normally sized fibers harbored variably sized non-rimmed, rounded, cytoplasmic, and subsarcolemmal vacuoles (Figures 2E,F). The latter stained positively with Sudan Black, demonstrating their lipid content (Figure 2G).

Transmission electron microscopy (TEM) further demonstrated the presence of atrophic fibers with a completely disrupted sarcomeric structure, intermingled with multiple and variably sized lipid droplets often surrounded by empty vacuoles (Figure 2H).

Respiratory workup performed for exertional dyspnea revealed a moderate restrictive ventilatory defect with an estimated 25% reduction in lung volumes. The total lung capacity was 92%, and the forced vital capacity (FVC) was 81% of the predicted value.

Whole-exome sequencing (WES) performed on DNA extracted from peripheral blood after informed consent and filtered using a multiexon amplicon panel containing a total of 230 genes linked to neuromuscular disorders and cardiomyopathies revealed a novel homozygous frameshift variant in exon 8 of the *PNPLA2* gene: NM_020376.4: c.1043del (p.Phe348SerfsTer18) (GenBank accession number PV231929), which was confirmed by Sanger sequencing. The patient’s parents were not available for segregation studies.

To rule out other contributing genetic factors, we further reviewed the exome data, identifying a heterozygous nonsense risk allele (NM_001011547.3:c.840G>A) in *SLC5A9*, which was previously associated with diabetic retinopathy (Ung et al., 2017). Additionally, we observed heterozygous variants of uncertain significance (VUS) in *FLAD1*, *MYH2*, and *DYSF*, though these were not linked to the patient’s phenotype. A heterozygous pathogenic variant (NM_170606.3:c.2961C>G) was also identified in *KMT2C*, a gene associated with autosomal dominant Kleefstra syndrome 2. However, the patient did not exhibit the corresponding phenotype; notably, a ClinVar report (accession SCV001362066.2) indicates that the region surrounding this variant is highly susceptible to pseudogene interference. No variants contributing to the phenotype were identified in the *ABHD5* gene.

**FIGURE 3:**
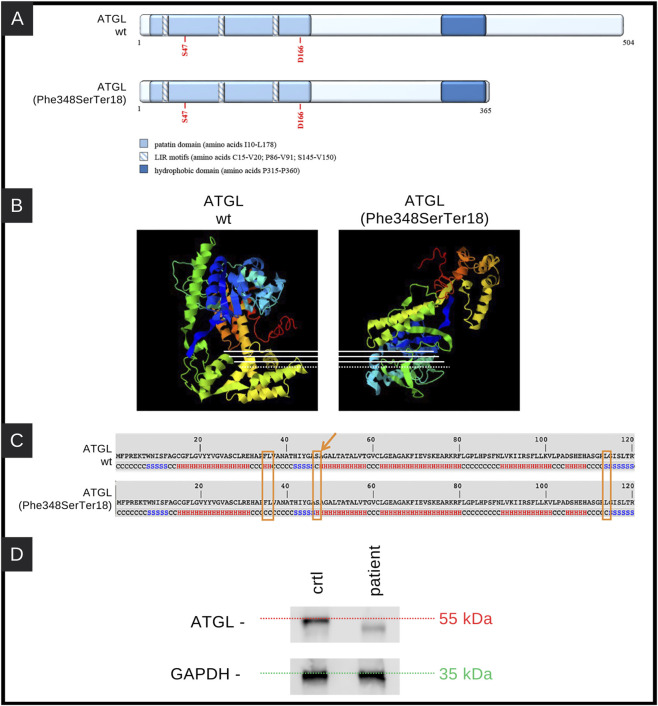
Identification and molecular characterization of the *PNPLA2* mutation. **(A)** Amino acid sequence alignment and secondary structure prediction for the N-terminal region (residues 1–120) of wild-type and mutant *ATGL*. The frameshift alters the conformation around Ser47, predicted to adopt an α-helical structure (arrow) instead of the native coiled-coil configuration. **(B)** Predicted tertiary structure of wild-type and mutant ATGL proteins. The mutant model shows conformational changes and overall destabilization, especially in the patatin domain, which contains the catalytic dyad required for TG hydrolysis. **(C)** Schematic representation of wild-type and mutant ATGL proteins. The mutant protein is truncated after residue 365, leading to the loss of the C-terminal region. Functional domains are indicated as patatin domain (amino acids 10–178), LIR motifs (C15–V20; P86–V91; S145–V150), and hydrophobic domain (P315–P360). **(D)** Western blot analysis of protein extracts from skeletal muscle tissue of the patient and a control subject. A truncated ATGL protein (∼40 kDa) was detected in the patient sample using an anti-ATGL polyclonal antibody (PA5-17436, Invitrogen). This is in contrast to the full-length protein (55 kDa) observed in the control sample. GAPDH, detected with an anti-GAPDH (226-335) mAb (H00002597-M01, Abnova), is shown as the loading control. The Western blot images were acquired using the C-DiGit® Blot Scanner (LICORbio, United States) and analyzed with Image Studio DiGit software (Ver 4.0). Contrast was uniformly adjusted in ImageJ software (National Institutes of Health). Original uncropped gels are available in Supplementary Figures S1, S2.

According to the ACMG criteria (Richards et al., 2015) and multiple *in silico* prediction tools (Franklin and ClinVar), the c.1043del (p.Phe348SerfsTer18) is a pathogenic class 5 variant and is predicted to cause a premature termination of ATGL (Figure 3A). *In silico* structural modeling using the I-TASSER platform indicated that the ATGL (p.Phe348SerfsTer18) undergoes conformational alterations within the patatin domain. Notably, the local secondary structure surrounding Ser47, the initial residue of the catalytic dyad, is predicted to shift from a coiled-coil to an α-helical conformation, likely impairing lipase function (Figure 3B). The overall folding of the mutant protein is predicted to be aberrant, supporting a deleterious impact on enzymatic activity (Figure 3C).

Western blotting performed on the patient’s muscle homogenate using the Anti-ATGL Polyclonal Antibody (PA5-17436, Invitrogen) showed the presence of a lower-molecular-weight band than that of the control, confirming the presence of a truncated ATGL protein. In addition to the likely impact on lipase activity, the variant also decreased protein stability, as evidenced by the weak signal in Western blot analysis (Figure 3D).

Beyond supportive care for his comorbidities—specifically diabetes, cataracts, and heart disease—no targeted treatment for NLSDM was initiated.

## 4 Discussion

NLSDM is an ultra-rare autosomal recessive disorder caused by biallelic mutations in the *PNPLA2* gene, which encodes ATGL, the key enzyme responsible for intracellular TG hydrolysis. To date, only 132 cases and 72 *PNPLA2* variants have been reported, and the natural history of the disease remains poorly characterized. No disease-modifying therapies are currently available, and clinical management is essentially supportive, aimed at alleviating symptoms and preventing complications. The extreme rarity of NLSDM and the phenotypic variability among the reported cases represent major barriers to the development of targeted therapies and standardized care protocols (Missaglia et al., 2017).

Our patient presented with a remarkably complex and multisystemic phenotype, including insulin-dependent diabetes, hypertrophic cardiomyopathy, progressive myopathy, and sensorineural hearing loss. This constellation of features reflects both the typical and the more atypical aspects of NLSDM, offering new insights into the clinical spectrum of the disease. Endocrine involvement, particularly diabetes mellitus, is not a universal feature in NLSDM, but it has been documented in up to 30% of cases, often in association with pancreatic steatosis and exocrine dysfunction (Samukawa et al., 2020). Our patient developed insulin-dependent diabetes at age 33, likely secondary to lipid accumulation in the pancreatic islets leading to β-cell dysfunction (Natali et al., 2013). Thus, our case further supports the inclusion of diabetes due to pancreatic dysfunction as a potential, though not obligatory, component of the NLSDM phenotype.

Cardiac involvement is a well-documented and potentially life-threatening complication of NLSDM, observed in approximately 40% of patients and encompassing both hypertrophic and dilated cardiomyopathies (Rajani et al., 2020). Our patient exhibited asymmetric left ventricular hypertrophy, mild right ventricular hypertrophy, and impaired systolic function. Notably, cardiac MRI revealed myocardial stress, and coronary angiography showed diffuse atheromatosis—a finding not systematically reported in NLSDM but potentially linked to the underlying lipid metabolism disorder (Hara et al., 2023). The exclusion of other genetic cardiomyopathies supports the causal role of PNPLA2 deficiency. The progressive hypertrophy, arrhythmias, and eventual contractile dysfunction seen in our patient are consistent with the natural course described in the literature.

Among hallmark features, Jordans’ anomaly—lipid-laden vacuoles in neutrophils—was evident in our patient (not shown) and remains a pathognomonic sign, described in 100% of the reported cases and readily identifiable in peripheral blood smears (Samukawa et al., 2020; Shahriyari et al., 2024), and the accessibility of the test makes it a key diagnostic clue with strong positive predictive value.

Less common manifestations included bilateral sensorineural hearing loss and cataracts—features more frequently associated with NLSDI but occasionally reported in NLSDM. Moreover, our patient displayed widespread, target-like skin lesions resembling prurigo. Given the non-contributory findings of the initial skin biopsy, a direct cutaneous involvement cannot be confirmed in this case. Nevertheless, cutaneous manifestations have been described in some patients with NLSDM, suggesting that dermatologic features may occur more frequently than traditionally recognized (Samukawa et al., 2020).

From a neuromuscular standpoint, the patient’s disease began with pain in the right shoulder, progressing to asymmetric limb and scapular girdle weakness, scapular winging (Figure 1), and frequent falls (Zheng and Liao, 2018). This pattern aligns with the typical presentation of NLSDM, which is often proximal, asymmetric, and associated with muscle atrophy—frequently more pronounced on the dominant right side (Kaneko et al., 2014). Muscle pain, although reported, is not usually a major complaint, and it was the first and disabling symptom in our case (Latimer et al., 2018).

Muscle MRI revealed a highly characteristic pattern of fatty replacement, with the predominant involvement of the gluteal, posterior thigh, and calf muscles (Figures 2A,B–C). The marked involution of the posterior tibialis with relative sparing of the anterior compartments mirrors the distribution described in Garibaldi et al. (2017). Additionally, a patchy pattern of fatty replacement in the soleus muscle—proposed as a disease signature—was observed, further confirming the diagnosis.

Myopathological analysis showed classical myopathic features, including vacuolization and marked lipid accumulation, as demonstrated by Sudan Black staining (Figures 2E,F–G). This lipid accumulation in the muscle biopsy was present in nearly all NLSDM patients, with frequencies of 98% in Samukawa M et al. Notably, rimmed vacuoles—occasionally observed in NLSDM—were absent in our patient (Shahriyari et al., 2024). Electron microscopy confirmed the presence of massive sarcomeric disorganization in atrophic fibers, with lipid storage (Figure 2H) (Shahriyari et al., 2024; Yang et al., 2024). The *bona fide* secondary myofibrillar disarray might contribute to the progressive muscle weakness developed by NLSDM patients.

Diagnosis was confirmed by the identification of a novel homozygous frameshift variant in *PNPLA2* (c.1043del; p.Phe348SerfsTer18), leading to premature truncation of ATGL within its catalytic patatin domain. This variant likely abolishes enzymatic function and compromises protein stability (Figure 3). There were no variants in *ABHD5* gene, supporting the concept of a broader phenotypic continuum between NLSDI and NLSDM. Interestingly, there was a nonsense heterozygous risk allele, NM_001011547.3:c.840G>A, in *SLC5A9*, previously associated with diabetic retinopathy (Ung et al., 2017), that could have an impact on the evolution of our patient’s diabetic retinopathy. Western blot analysis of patient-derived muscle tissue revealed the presence of a truncated ATGL protein, which is consistent with the predicted premature termination of translation (Figure 3D). Some truncating variants located in the C-terminal domain of ATGL might still preserve lipase activity due to the presence of the catalytic site in the N-terminal portion of the enzyme (Kobayashi K. et al., 2008). In this regard, the demonstration of altered tertiary conformation and the reduced steady state levels of the protein observed in the Western blot analysis likely account for the lipid accumulation in this and potentially in other NLSDM cases (Fischer et al., 2007; Kobayashi et al., 2008). This highlights the importance of further investigation to correlate the genotype with protein expression and disease severity.

Furthermore, bioinformatics analysis displayed conformational changes in the mutant ATGL protein, particularly within the patatin domain, which harbors the catalytic dyad essential for TG hydrolysis. Indeed, in the mutant protein model, Ser47 is predicted to adopt an α-helical conformation instead of the native coiled-coil structure (Figure 3B). This structural shift may interfere with the catalytic machinery, possibly abolishing enzymatic activity. Moreover, the overall tertiary structure of the mutant protein appears to be destabilized, suggesting broader folding defects that may further hinder proper protein localization or interaction with coactivators such as CGI-58, known to be essential for ATGL activation. These structural insights reinforce the hypothesis that the c.1043del variant exerts its pathogenic effects through a combination of premature truncation, disruption of the catalytic domain, and global misfolding.

This report describes the first documented case of NLSDM associated with a novel homozygous frameshift mutation in *PNPLA2* (c.1043del; p.Phe348SerfsTer18), thereby expanding both the genetic and phenotypic spectra of the disease. The patient’s multisystemic involvement—including neuromuscular, metabolic, cardiac, auditory, and ophthalmologic features—illustrates the considerable clinical variability of NLSDM and suggests a broader overlap with NLSDI than previously recognized. A major contribution of this work lies in the molecular demonstration, via Western blot analysis, of a truncated ATGL isoform in patient-derived muscle tissue. This is the first report to confirm the expression of this specific truncated protein in human muscle, providing direct evidence of the mutation’s pathogenic impact at the protein level. Although most severe NLSDM cases lack detectable ATGL, our findings highlight the importance of integrating genetic, structural, and functional data to better define the genotype–phenotype correlations.

NLSDM currently lacks specific treatment. Supportive therapies can generally improve the consequences of damage to internal organs, especially with regard to cardiomyopathy.

Many patients have been on a special diet that is poor in long-chain fatty acids and enriched with medium-chain fatty acids for years, but no positive effects were reported (Angelini et al., 2019; Missaglia et al., 2022). Some therapies, which could reduce lipid storage, such as bezafibrate, a peroxisome proliferator-activated receptor (PPAR) agonist, have been tested in a limited number of NLSDM patients (two patients and one heterozygous carrier) but did not result in significant clinical advantages (Van De Weijer et al., 2013; Garcia et al., 2018). However, bezafibrate treatment has benefited these patients by reducing lipid accumulation and ameliorating fat oxidative capacity. Therefore, the lack of clinical changes could be due to the relative short duration of the treatment (6 months) or to the fact that treatment was initiated in patients at a later stage of the disease. Longer treatments in a greater number of patients would be required to verify the real efficacy of the therapeutic intervention.

Recently, Zhang et al. (2025) used an adeno-associated virus 9 (AAV9) vector to deliver the control sequence of *PNPLA2* gene in a murine model with an *ATGL* mutation, mimicking arrhythmogenic cardiomyopathy (ACM) phenotypes (arrhythmias, lipid accumulation, and fibrosis), and demonstrated that early intervention prevented ACM onset, whereas later treatment rescued clinical symptoms, extending survival. These findings highlight that AAV9-mediated *PNPLA2* gene supplementation could be a promising therapeutic strategy for the treatment of NLSDM.

## 5 Patient perspective

The patient expressed a growing sense of frustration due to the progressive nature of his disability. He described significant neurosensory difficulties, especially hearing loss and impaired vision from diabetic retinopathy, which severely affected daily activities. However, his greatest concern remained the marked muscle weakness, which has led to a substantial loss of independence and poses a constant risk of falling, impacting his ability to work. He reported persistent anxiety related to his cardiac problems, which he recognized as an additional and unpredictable complication in his clinical course. Receiving a definitive autosomal recessive genetic diagnosis was described as a moment of relief: it closed a long and tiring diagnostic journey and offered important reassurance regarding reproductive risks for his family.

## Data Availability

The datasets presented in this article are not readily available because of ethical and privacy restrictions. Requests to access the datasets should be directed to the corresponding author.

## Supplementary material

SUPPLEMENTARY VIDEO S1 Video demonstrating the patient’s waddling (myopathic) gait, with noticeable reduction of pendular movement of the right upper limb.

SUPPLEMENTARY FIGURE S1 Original uncropped images of Western blot analyses for ATGL.

SUPPLEMENTARY FIGURE S2 Original uncropped images of Western blot analyses for GAPDH, used as control.
