# Supplementary figures and images for: Case Report: a novel PNPLA2 homozygous frameshift variant causing severe neutral lipid storage disease with myopathy (NLSDM) in a Moroccan patient
Source: Front Genet. 2026 May 26;17:1701218. doi: 10.3389/fgene.2026.1701218 (PMC13245935; doi:10.3389/fgene.2026.1701218)

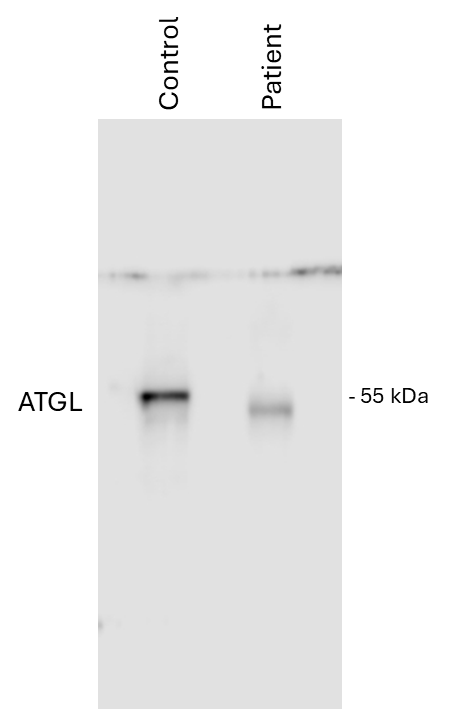


Figure S1: Original uncropped images of Western blot analyses for ATGL.

Supplement: Supplementary file 1 [file Supplementaryfile1.docx]

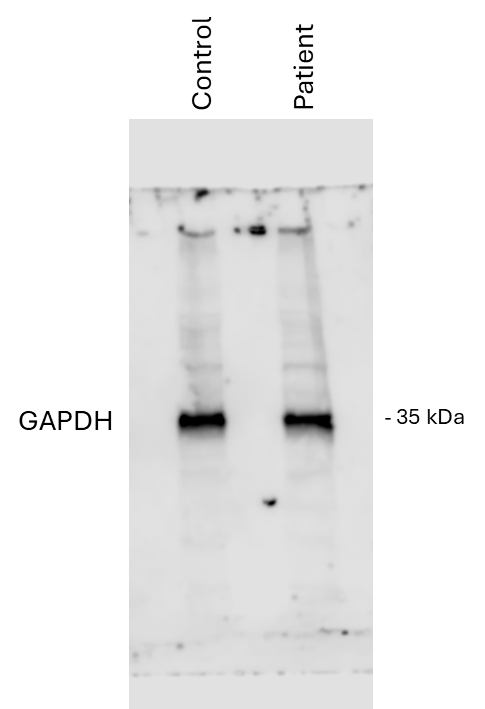


Figure S2: Original uncropped images of Western blot analyses for GAPDH.

Supplement: Supplementary file 2 [file Supplementaryfile2.docx]
